# Supplementary material for: ISME—incoherent sampling of multi‐echo data to minimize cardiac‐induced noise in brain maps of R2* and magnetic susceptibility
Source: Magn Reson Med. 2025 Sep 17;95(2):897–911. doi: 10.1002/mrm.70087 (PMC12681315; doi:10.1002/mrm.70087)
Supplement: Supplementary file 1 — Figure S1. (A) Motion degradation index (MDI) of the data sets acquired with the standard multi‐echo trajectory and incoherent sampling of multi‐echo data (ISME). The average MDI from Subject 1 was 4.5 ± 0.8 s−1, and the average MDI among the other subjects was 3.3 ± 0.3 s−1. (B) R2* map computed from the first repetition of the standard multi‐echo trajectory for Subject 1 (MDI = 4.96 s−1) and for 1 participant with MDI = 3.71 s−1. Figure S2. Eye movement during the acquisition of MRI data leads to image aliasing along the slow phase‐encoding direction of a three‐dimensional (3D)–encoded image (blue arrows). This aliasing is present in data acquired with a standard multi‐echo trajectory (A) but not with incoherent sampling of multi‐echo data (ISME) (B). Figure S3. (A) Change in the Akaike information criterion (AIC) with the order of the Taylor expansion of the signal decay (M). (B) Increase of the evidence lower bound (ELBO) with the order of the polynomial model of the dependence of image noise on the echo time of the data (N), compared with N = 0 (uniform noise level across echo times), for standard multi‐echo trajectory and incoherent sampling of multi‐echo data (ISME). (C) Echo‐specific weights computed for different polynomial orders (M = 2). [file MRM-95-897-s001.docx]

# ISME - Incoherent Sampling of Multi-Echo data to minimize cardiac-induced noise in brain maps of R_2_* and magnetic susceptibility

Quentin Raynaud^1*^, Rita Oliveira^1*^, Nadège Corbin^2^, Yaël Balbastre^3^, Ruud B. van Heeswijk^4^, Antoine Lutti^1^

*^1^Laboratory for Research in Neuroimaging, Department for Clinical Neuroscience, Lausanne University Hospital and University of Lausanne, Lausanne, Switzerland*

*^2^Centre de Résonance Magnétique des Systèmes Biologiques, UMR5536, CNRS/University Bordeaux, Bordeaux, France*

*^3^Department of Experimental Psychology, University College London, London, United Kingdom*

*^4^Department of Diagnostic and Interventional Radiology, Lausanne University Hospital and University of Lausanne, Lausanne, Switzerland.*

*These authors contributed equally to this work.

**Correspondence**

Antoine Lutti

Laboratory for Research in Neuroimaging, Department for Clinical Neuroscience, Lausanne University Hospital, Ch. de Mont-Paisible 16, CH-1011 Lausanne

Email: [antoine.lutti@chuv.ch](mailto:antoine.lutti@chuv.ch)

# Supplementary material

## Motion degradation index

The index introduced in Castella et al. ^1–3^ was calculated from each multi-echo dataset to estimate the level of image degradation caused by head motion (‘Motion Degradation Index’, MDI). The distribution of MDI values peaked at MDI ~3.5s^-1^, reflecting an overall high image quality (Figure S1). However, four datasets from the same subject exhibit MDI values above 4s^-1^, indicative of poor quality due to head motion. The data from this subject was removed from subsequent analyses.

## Mitigation of eye movement artefacts

Eye movement during the acquisition of MRI data leads to image aliasing along the slow phase-encoding direction of a 3D-encoded image. When the slow phase-encoding direction is oriented along the anterior-posterior direction of the patient, this aliasing propagates across the brain with a standard multi-echo trajectory (Figure S2A). However, this aliasing artifact is largely mitigated with the proposed ISME approach (Figure S2B). To keep the focus of this study specifically on cardiac-induced effects, the imaging box was tilted by 30 degrees in the sagittal plane to displace this artefact below the brain.^4^

## Model optimization for estimation of R2* using NWLS

Fitting a nonlinear model with a weighted LS approach involves two sets of unknowns: the model parameters and the weights, with the latter corresponding to the inverse of the variance of the residuals at each echo time. Estimating both sets of parameters by maximum-likelihood results in biased estimates of (co)variance, which in turn bias the estimated model parameters. In contrast, restricted maximum-likelihood approaches (ReML) yield unbiased estimates of variance. ReML, however, does not lend itself easily to nonlinear models. We therefore devised a two-step procedure, whereby a linear ReML estimate of variance is performed first using a Taylor expansion of the transverse signal decay, and the resulting weights are then used in a nonlinear weighted least square (NWLS) fit. ReML estimation was performed using the function `spm_reml` in SPM (the analysis script is provided with this article). The covariance matrix of the residual noise was assumed diagonal, with weights modelled as a polynomial in TE. As is standard in SPM, we assumed that this covariance matrix is shared across all voxels within a brain volume.

We identified the optimal order of the Taylor expansion (M) from the Akaike information criterion corrected for small sample size (AICc). The AICc rewards the goodness of fit and penalizes the number of estimated parameters.^5^ The minimal values of the AICc were found for Taylor expansion orders of 1 and 2 (Figure S3A). We opted for a Taylor expansion of order M=2 to accommodate local deviations from the linear approximation of the signal decay due to e.g. high decay rate in iron-rich regions (pallidum, red nuclei, substantia nigra,..) or deviations from the exponential behaviour in white matter and sub-cortical areas.^6–8^

From the results of the NLS analyses shown in Figure 5C, models of the dependence of the residuals on the echo time of the data were assumed to follow a polynomial form of order N. Model selection was conducted from the estimates of the evidence lower bound (ELBO) provided by the implementation of ReML of the SPM software.^9^ The ELBO favours the reduction of residual errors and penalises model complexity. An optimal noise model maximises the ELBO. Compared to N=0 (uniform residual level across echo times), the optimal noise model was found to be N=3 for data acquired with the standard multi-echo trajectory. The corresponding increase in ELBO was ΔELBO=ELBO_N=3_-ELBO_N=0_=43.3 (Figure S3B). With the proposed ISME method, the optimal polynomial order was N=4. The corresponding increase in ELBO (ΔELBO=ELBO_N=4_-ELBO_N=0_=73.1) is higher than in data acquired with the standard multi-echo trajectory. Image-specific weights were computed as the inverse of the diagonal elements of the noise covariance matrix estimated by ReML. The dependence of the weights on the echo time of the data shows little change for values of N≥4 (Figure S3C).

# Supplementary figures


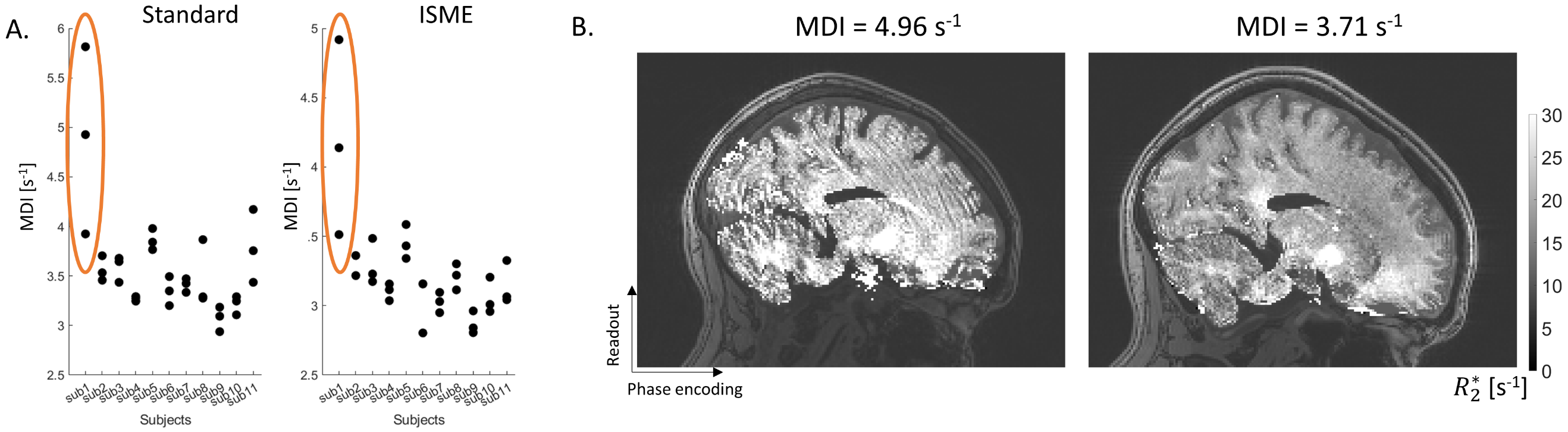


Figure S1: (A) Motion degradation index (MDI) of the datasets acquired with the standard multi-echo trajectory and ISME. The average MDI from subject 1 was 4.5±0.8s^-1^ and the average MDI amongst the other subjects was 3.3±0.3s^-1^. (B) R_2_* map computed from the first repetition of the standard multi-echo trajectory for subject 1 (MDI=4.96s^-1^) and for one participant with MDI=3.71s^-1^.


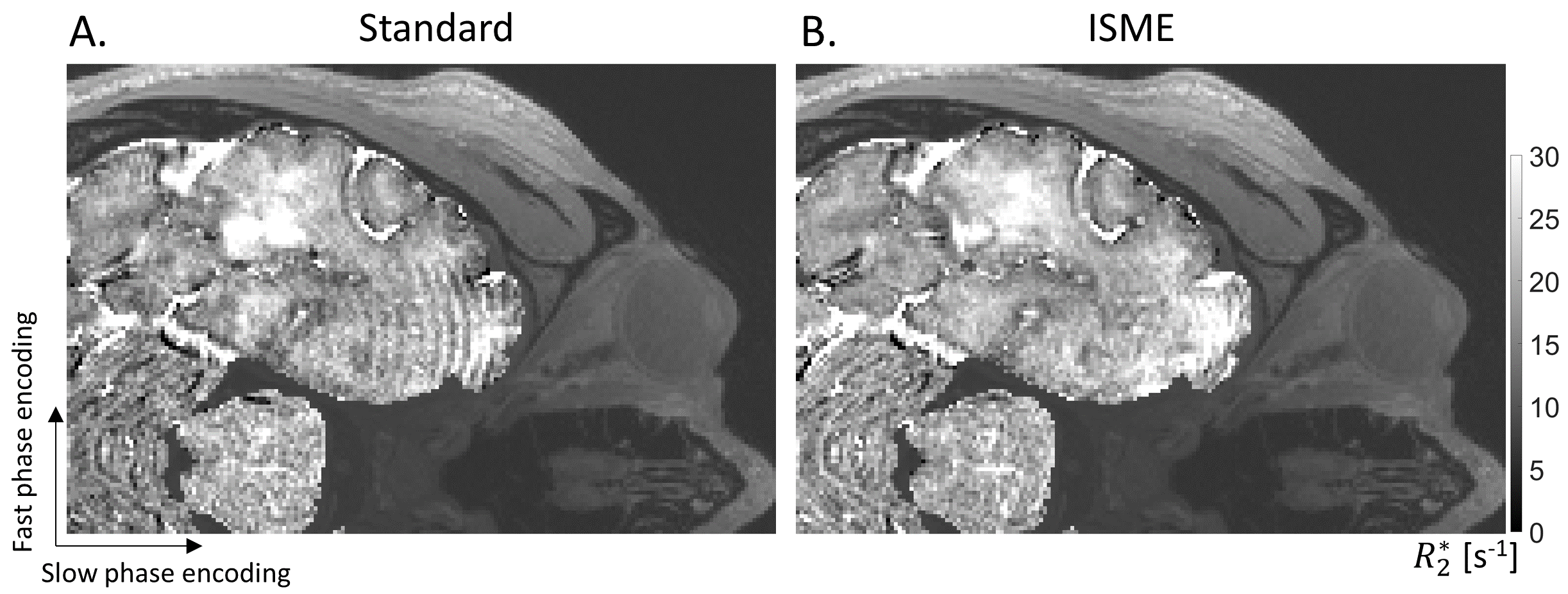


Figure S2: Eye movement during the acquisition of MRI data leads to image aliasing along the slow phase-encoding direction of a 3D-encoded image (blue arrows). This aliasing is present in data acquired with a standard multi-echo trajectory (A) but not with ISME (B).


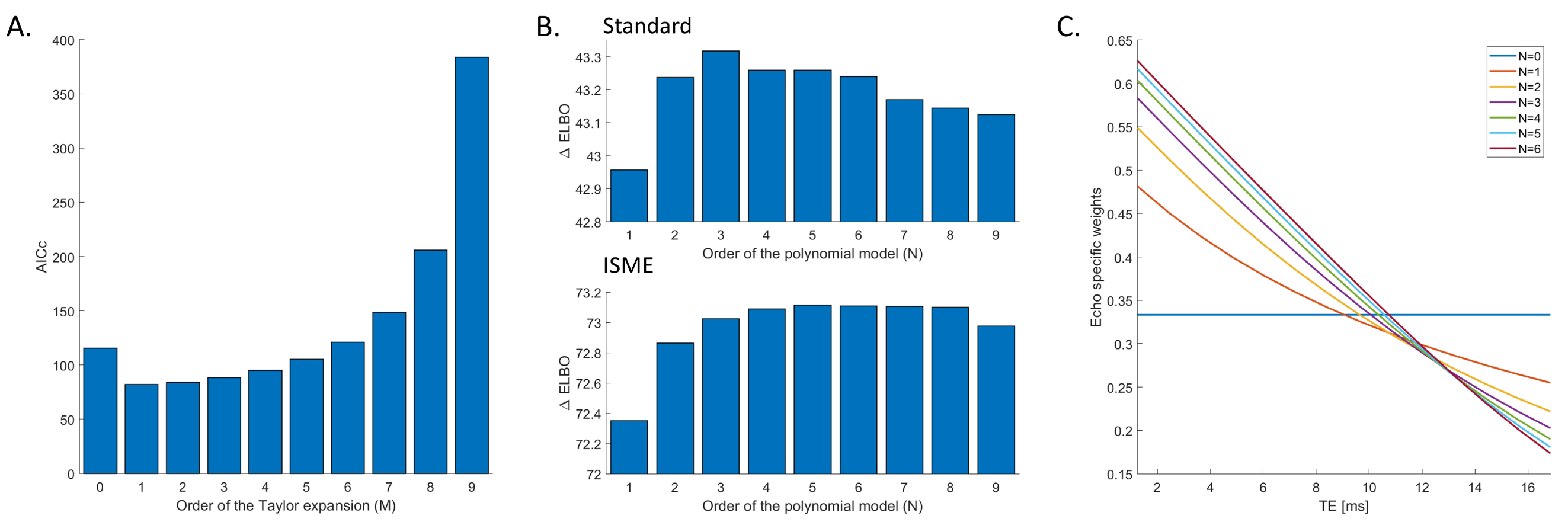


Figure S3: (A) Change in the Akaike information criterion (AICc) with the order of the Taylor expansion of the signal decay (M). (B) Increase of the evidence lower bound (ELBO) with the order of the polynomial model of the dependence of image noise on the echo time of the data (N), compared to N=0 (uniform noise level across echo times), for standard multi-echo trajectory and ISME. (C) Echo specific weights computed for different polynomial orders (M=2).

# Reference

1. Castella R, Arn L, Dupuis E, Callaghan MF, Draganski B, Lutti A. Controlling motion artefact levels in MR images by suspending data acquisition during periods of head motion. *Magnetic Resonance in Medicine*. 2018;80(6):2415-2426. doi:10.1002/mrm.27214

2. Lutti A, Corbin N, Ashburner J, et al. Restoring statistical validity in group analyses of motion-corrupted MRI data. *Human Brain Mapping*. 2022;43(6):1973-1983. doi:10.1002/hbm.25767

3. Corbin N, Oliveira R, Raynaud Q, et al. Statistical analyses of motion-corrupted MRI relaxometry data computed from multiple scans. *Journal of Neuroscience Methods*. 2023;398. doi:10.1016/j.jneumeth.2023.109950

4. Budrys T, Veikutis V, Lukosevicius S, Gleizniene R, Monastyreckiene E, Kulakiene I. Artifacts in magnetic resonance imaging: How it can really affect diagnostic image quality and confuse clinical diagnosis? *Journal of Vibroengineering*. 2018;20(2):1202-1213. doi:10.21595/jve.2018.19756

5. Cavanaugh JE, Neath AA. The Akaike information criterion: Background, derivation, properties, application, interpretation, and refinements. *Wiley Interdisciplinary Reviews: Computational Statistics*. 2019;11(3). doi:10.1002/wics.1460

6. Oliveira R, Raynaud Q, Jelescu I, Kiselev VG, Kirilina E, Lutti A. In Vivo Characterization of Magnetic Inclusions in the Subcortex From Nonexponential Transverse Relaxation Decay. *NMR in Biomedicine*. 2025;38(6). doi:10.1002/nbm.70051

7. Chan KS, Chamberland M, Marques JP. On the performance of multi-compartment relaxometry for myelin water imaging (MCR-MWI) – test-retest repeatability and inter-protocol reproducibility. *NeuroImage*. 2023;266. doi:10.1016/j.neuroimage.2022.119824

8. Chen WC, Foxley S, Miller KL. Detecting microstructural properties of white matter based on compartmentalization of magnetic susceptibility. *NeuroImage*. 2013;70:1-9. doi:10.1016/j.neuroimage.2012.12.032

9. Friston KJ, Penny W, Phillips C, Kiebel S, Hinton G, Ashburner J. Classical and Bayesian inference in neuroimaging: Theory. *NeuroImage*. 2002;16(2):465-483. doi:10.1006/nimg.2002.1090
